# Education as a tool to modify anti-obesity bias among Pediatric residents

**DOI:** 10.5116/ijme.58b1.46e3

**Published:** 2017-03-11

**Authors:** Marielisa Rincon-Subtirelu

**Affiliations:** 1 Children's Mercy Hospital and Clinics, Department of Pediatrics, Division of Pediatric Endocrinology, USA

**Keywords:** Education, anti-obesity bias, Pediatric residents, USA

## Introduction

Bias can affect the care of certain groups, including obese patients.^1^ The classic stereotype about obese people is encountered in healthcare. One study found that health care professionals demonstrate strong negative associations toward obese persons both with attitude and stereotype measures.^2^ Obesity specialists are not any different, showing a pro-thin, anti-fat implicit bias, in addition to acceptance of stereotypes of lazy, stupid, and worthless.^3^ A cross-sectional survey to internal medicine residents demonstrated that 80% of residents reported feeling uncomfortable examining obese patients or difficulty feeling empathy for them.^4^

There are no studies evaluating the presence of bias exclusively in pediatricians or in pediatric residents. Moreover, there are no studies evaluating how to change anti-obesity bias in pediatric residents.  The purpose of this paper is to describe how we found answers to these queries, first, by confirming the presence of anti-obesity bias in pediatric residents and, second, by evaluating the efficacy of an obesity curriculum to modify any initial bias.

### Implicit and explicit anti-obesity bias in health care

The Implicit Association Test (IAT), a tool designed to demonstrate implicit bias, has been used to demonstrate implicit weight bias among health care students and practitioners,^5^^,^^6^ even those specializing in obesity.^3^  An analysis of IATs done by physicians found strong anti-fat bias.^6^  This bias has a detrimental effect on the delivery and access to care for these patients, ultimately affecting their health.^7^

A small group of pediatric residents were evaluated for their explicit and implicit anti-obesity bias. The initial IAT average indicated strong to moderate automatic preference for thin people. In addition, there was evidence of explicit bias based on questionnaire responses. This was more evident in the senior resident class.

### Changing anti-obesity bias with education

An important aspect of changing bias is to recognize its presence.  A study revealed that over one third of medical students at one university had implicit anti-fat bias, but most were not aware of the bias.^5^  MacLean et al. suggested that one way to decrease anti-obesity bias in health care was to train medical staff about stereotyping, obesity and obese people.^8^

There is evidence that bias may be even greater long after medical school and residency training have been completed. A study reviewing bias in primary care providers found that those with more years of practice had greater feelings of dislike towards the obese. The authors proposed that ongoing continuing education on obesity and bias should be provided particularly to those who have been in practice the longest.^9^ This is important because it implies that education received during training is more likely to have greater impact.

Early interventions could modify bias, in favor of better health care for the obese. To date, there is no study demonstrating that education can modify implicit bias in pediatric residents. 

An obesity curriculum was designed and implemented to understand the impact of education.  The curriculum was provided as a lecture series, reading material and video. The lecture series was divided into four 30-minute lectures on obesity diagnosis, management, and bias. A second IAT was administered 6 weeks after completion of the curriculum, demonstrating that there was significant improvement from pre to post IAT scores.  The participants were also invited to take part in a group discussion once the second IAT and the lecture series were completed. The residents stated that awareness of their bias made them more likely to be mindful of their own attitude during a clinical encounter.  Those who reported anti-obesity bias stated that this bias was likely due to their own history of obesity or due to frustration with patients on their lack of success with weight loss.

### Conclusions and implications

Anti-obesity bias is undeniably present among pediatric residents. Acknowledging the existence of anti-obesity bias in healthcare is the first step towards change.^5^ The presence of bias in pediatric residents is comparable to that encountered among other groups of healthcare providers.^2^ The anti-obesity bias is more explicit as the residents advance in training. This observation suggests that interventions to positively impact anti-obesity bias should be incorporated as early as possible in medical training.

Educational interventions can decrease anti-obesity bias. This confirms the value of obesity education, and provides an argument to pediatric residency training programs towards dedicating time and resources to this endeavor.

The ultimate goal of understanding anti-obesity bias should be to provide state of the art, timely, and compassionate care to all patients with obesity. Future studies may need to link changes in pediatric residents’ bias with patient satisfaction and health care cost and outcomes.

### Acknowledgements

I wish to thank Kimberly Reid, Ellen Tessmann, Lisa A. Holstrom, and Robert Harper for their review and suggestions in the preparation of this manuscript.

### Conflicts of Interest

The author declares that they have no conflict of interest.
